# Effect of different geometrical structure of scapula on functional recovery after shoulder arthroscopy operation

**DOI:** 10.1186/s13018-019-1362-z

**Published:** 2019-09-14

**Authors:** Xuchao Shi, Yuanlin Xu, Bo Dai, Weilong Li, Zhennian He

**Affiliations:** Department of Orthopaedics Surgery of Beilun People’s Hospital, No. 1288, Lushan East Road, Ningbo, 315800 Zhejiang Province China

**Keywords:** Critical shoulder angle, Acromion index, Glenoid inclination, Functional recovery

## Abstract

**Background:**

There are no published studies of the influence of geometry of the scapula on the postoperative recovery of rotator cuff injuries. Our aim was to explore the relationship between the critical shoulder angle (CSA), acromion index (AI), glenoid inclination (GI), and postoperative repair outcomes in shoulder joints after arthroscopic supraspinatus tendon repair.

**Methods:**

Sixty two patients suffering a supraspinatus tear were analyzed retrospectively following failure of conservative treatment and subsequent shoulder arthroscopy in our hospital. Standard anterior and posterior X-rays of the injured shoulder had been performed prior to surgery, with follow ups for at least 2 years (24–43 months). Magnetic resonance imaging (MRI) was performed 2 years after surgery to assess repair of the supraspinatus tendon. Patients were divided into either the intact or re-tear group, according to the MRI results. In addition, assessments using the Constant Shoulder Score (CSS), the American Shoulder and Elbow Surgeon (ASES) Shoulder Assessment Form, the University of California at Los Angeles (UCLA) score and visual analog scale (VAS) score were performed to establish shoulder function at the 2-year evaluation for each patient.

**Results:**

The mean CSA of all patients was 35.79° ± 3.59°, mean AI was 0.72 ± 0.05, and mean GI was 15.87° ± 3.62°. The CSA, AI, and GI in the intact group were statistically significantly different than the re-tear group (*p* < 0.05). There was no correlation between the magnitude of the CSA, AI, or GI and any shoulder function score (*p* > 0.05).

**Conclusions:**

The geometry of the scapula had no significant effect on the recovery of postoperative function of patients with rotator cuff injury, but the value of the CSA, AI, and GI affected the risk of rotator cuff re-tear.

## Background

Tearing of the rotator cuff is a common injury of the shoulder joint. Its pathogenesis is related to many factors. Many reports have been published that demonstrate that the risk of such injuries and recurrence post-repair are related to age, smoking, initial tear size, and fatty infiltration [1–6]. Recent studies [4–7] have demonstrated that differences in scapula geometry also affect the incidence of rotator cuff tears (RCTs), including the critical shoulder angle (CSA), acromion index (AI), or glenoid inclination (GI).

**Fig. 1:**
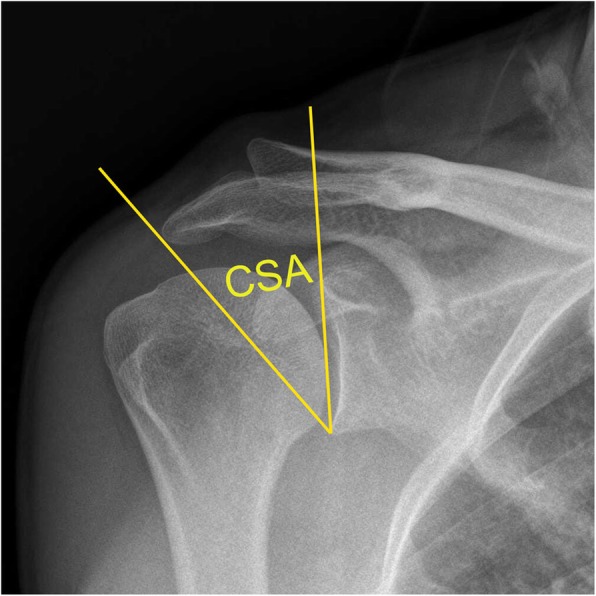
The critical shoulder angle (CSA) is the angle formed from a line parallel to the glenoid where it crosses a line from the most inferior part of the glenoid and the most lateral edge of the inferior acromion

The AI is obtained by measuring the distance from the glenoid plane to the lateral border of the acromion (GA) divided by the distance from the glenoid plane to the lateral aspect of the humeral head (GH) [2]. The CSA is a combination of AI and GI [7, 8]; it is the angle formed from a line parallel to the glenoid where it crosses a line from the most inferior part of the glenoid and the most lateral edge of the inferior acromion (Fig. 1). Studies have shown that having a CSA > 35° is associated with RCTs, while a CSA < 30° is associated with osteoarthritis of the glenohumeral joint [9–12]. In order to establish the relationship between CSA and RCTs, researchers have previously conducted studies on biomechanics. Gerber et al. [13] demonstrated that load and compressive forces on the supraspinatus tendon increased significantly when the CSA > 38°, compared with CSAs < 38°. In addition, Moor et al. [7] demonstrated that when CSA > 35°, stress on the shoulder joint was significantly altered, resulting in an increased risk of humeral translation. Previous studies have demonstrated that the size of the AI facilitates RCTs [14–16]. Although many publications have discussed the impact of various geometries of the scapula on RCTs, no study has convincingly compared the effects of CSA on postoperative repair of a rotator cuff tears (RCT). MRI is a non-invasive tool that can readily observe changes in the integrity of tendons and muscle atrophy after repair of an RCT.

The main purpose of our study was to investigate the effects of different values of CSA, AI, and GI on repair of the supraspinatus after an RCT, with evaluation of the effects of these parameters through the clinical performance of the shoulder joint after surgery.

## Methods

### Patient selection

Patients diagnosed with a simple supraspinatus tendon tear from April 2010 to June 2014, who had failed conservative treatment and had therefore undergone shoulder arthroscopy in our hospital were selected. The study was approved by the local institutional review board. All patients provided written informed consent prior to allocation into the study. Our inclusion criteria for patients were (1) symptomatic patients with a full-thickness supraspinatus tear evaluated through shoulder ultrasound or MRI, for whom conservative treatment had failed and (2) preoperative anterior and posterior shoulder X-rays were performed. Exclusion criteria were (1) multiple tears of the rotator cuff except of the supraspinatus, (2) history of shoulder surgery, and (3) presence of degenerative or neuromuscular diseases of the shoulder joint.

### Surgical technique

All shoulder surgery was performed by a senior surgeon (HZN). All patients underwent general anesthesia and were placed in a beach chair position, which reduced blood pressure, with systolic blood pressure controlled at approximately 95 mmHg intraoperatively. Using a posterior approach with an arthroscope, the anterior joint capsule, the labrum, the rotator cuff, the glenoid, and the cartilage of the humeral head were explored with the biceps muscle tendon as the starting point. An anterior approach assessed the labrum and posterior capsule and explored the subacromial gap. After exploration, capsulotomy was performed to remove any edema or hypertrophic synovial tissue in the glenohumeral joint cavity. During this intervention, free the rotator cuff to prevent the tension from being too high when it was pulled to the position where it needs to be fixed. A standard rotator cuff repair was then performed using a double row technique: 2 threaded suture anchors were placed through the rotator cuff, 45° to the humerus, with the rotator cuff suspended from the articular surface.

### Postoperative rehabilitation

Shoulder position was immobilized in a neutral position for the 4 weeks following surgery. Gentle passive functional exercise started on the first day after surgery, with internal and external rotation activities or a pendulum activity performed without pain. Active exercise began after 2 weeks, with active flexion to positions higher than the shoulder joint generally starting 4–6 weeks after surgery. At this time, strength training of the shoulder joint was initiated, with full recovery in levels of activity taking 3–6 months.

### Patient assessment

**Fig. 2:**
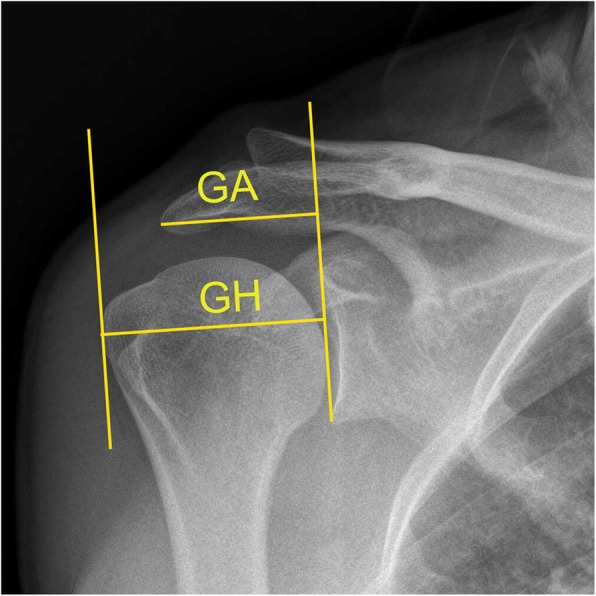
The acromion index (AI) is obtained by measuring the distance from the glenoid plane to the lateral border of the acromion (GA) divided by the distance from the glenoid plane to the lateral aspect of the humeral head (GH)

**Fig. 3:**
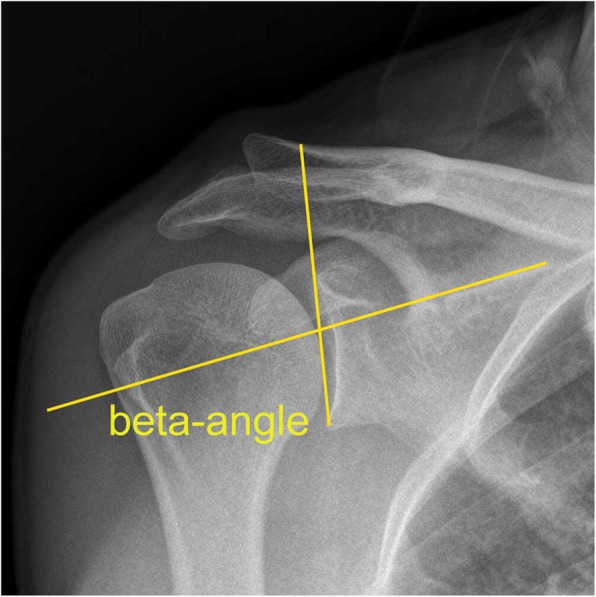
The glenoid inclination (GI) is formed by the intersection of a line passing through the floor of the supraspinatus fossa and the line connecting the upper and lower glenoid

Initial assessment of the patient was performed using a shoulder X-ray. The CSA, AI, and GI were measured by two physicians (SXC, XYL) with the order in which they were measured randomized. The CSA is the angle formed by the intersection of a line connecting the upper and lower edges of the glenoid and a line connecting the outermost point of the shoulder to the lower edge of the glenoid (Fig. 1) [7, 8, 17]. To measure AI, the GA and GH were measured separately, with AI = GA/GH (Fig. 2) [7]. The beta angle is formed by the intersection of a line passing through the floor of the supraspinatus fossa and the line connecting the upper and lower glenoid (Fig. 3) [18]. GI is calculated as 90° minus the beta angle.

A functional assessment of the shoulder was performed 2 years after surgery, using Constant Shoulder Score (CSS), American Shoulder and Elbow Surgeon (ASES), and UCLA scores. The VSA score was used to assess postoperative shoulder pain, using a pain score from 0 to 10, with 0 indicating no pain at all and 10 indicating the most severe pain the patient could imagine.

At the 2-year follow-up, patients also underwent a shoulder examination using a 1.5-T MRI. Postoperative cuff integrity was classified into 5 categories using oblique coronal, oblique sagittal, and transverse views of T2-weighted images: Type I, the repaired rotator cuff appeared to be of a thickness on each image that would have uniform low strength compared to a normal cuff; Type II, thick enough compared with a normal cuff associated with some high-intensity areas; Type III, cuff thickness less than half of the normal cuff, but with no discontinuity, indicating partial thickness tearing; Type IV, only a slight discontinuity in 1 or 2 slices on the slanted coronal and sagittal images, indicating a small full-thickness tear; V-type, major discontinuities in more than 2 slices observed on the oblique coronal and sagittal images, indicating a medium or large full-thickness tear [19]. Repair integrity was classified into two categories: intact or torn (full thickness re-tear, Sugaya Type IV or V [2]).

### Statistical analysis

Statistical analysis was performed using SPSS 22.0 (IBM Corporation, Armonk, NY, USA). All count data were described in terms of mean and SD and compared using an independent-samples *t* test. All measurements were compared using a chi-square test. A Pearson correlation was used to assess the relationship between CSA, AI, GI, and shoulder function scores.

Inter- and intra-observer reliability were quantified using the intraclass correlation coefficient (ICC), which ranges from 0 to 1.0, with higher values indicating better reliability. An ICC of 0.0–0.25 indicating little correlation, 0.26–0.49 indicating low correlation, 0.50–0.69 indicating moderate correlation, 0.70–0.89 indicating high correlation, and 0.90–1.00 indicating very high correlation.

## Results

### Demographics

A total of 62 patients were followed up. Mean follow-up time was 34 ± 4 months (24–43 months). MRIs revealed that 34 patients had an intact rotator cuff 2 years after surgery, while 28 patients had rotator cuff re-tears. The demographic characteristics of all patients are shown in Table 1. No significant difference existed in the demographic characteristics of the intact group compared with the re-tear group (*p* > 0.05).

**Table 1:** Different characteristics between intact group and re-tear group

	Intact group (*n* = 34)	Re-tear group (*n* = 28)	*p*
Age	52.76 ± 6.38	51.14 ± 6.40	.324
BMI	22.38 ± 2.75	21.86 ± 2.79	.460
Sex			
Male	16	17	.284
Female	18	11	
Side			
Left	9	7	.895
Right	25	21	
CSA	34.74 ± 3.25	37.07 ± 3.40	.008
AI	0.71 ± 0.04	0.74 ± 0.05	.006
GI	16.82 ± 3.51	14.71 ± 3.46	.021
CSS	73.35 ± 6.15	76.04 ± 5.83	.085
ASES	87.68 ± 4.37	87.53 ± 5.47	.911
UCLA	29.65 ± 2.77	30.57 ± 2.82	.200
VAS	1.32 ± 1.07	1.64 ± 1.39	.311

*BMI* body mass index, *CSA* critical shoulder angle, *AI* acromial index, *GI* glenoid inclination, *CSS* Constant Shoulder Score, *ASES* American Shoulder and Elbow Surgeon, *UCLA* University of California at Los Angeles, *VAS* visual analog scale

### Radiographic measurement

The CSA, AI, and GI were measured for each patient. Mean CSA was 35.79° ± 3.59° for all patients, mean AI was 0.72 ± 0.05, and mean GI was 15.87° ± 3.62°. It can be seen from Table 1 that both the CSA and AI in the re-tear group were significantly larger than in the intact group (*p* < 0.05). However, in the intact group, the GI was significantly larger than in the re-tear group (*p* < 0.05).

### Result score

Functional shoulder assessments were also evaluated on patients 2 years after surgery, including CCA, ASES, UCLA, and VAS scores. As can be seen from Table 2, no significant correlation existed between the values of CSA, AI, or GI with any shoulder joint evaluation indicator (*p* > 0.05). In addition, in Table 1, there was no difference in shoulder function in the intact group compared with the re-tear group (*p* > 0.05).

**Table 2:** Relationship between the result of radiographic measurement and functional scores of shoulder joint 2 years after operation

	CSA		AI		GI	
	*r*	*p*	*r*	*p*	*r*	*p*
CSS	0.23	.063	0.09	.475	0.22	.073
ASES	− 0.02	.887	0.19	.140	− 0.08	.536
UCLA	− 0.17	.168	0.05	.707	− 0.11	.384
VAS	− 0.15	.258	0.15	.230	0.19	.136

*CSA* critical shoulder angle, *AI* acromial index, *GI* glenoid inclination, *CSS* Constant Shoulder Score, *ASES* American Shoulder and Elbow Surgeon, *UCLA* University of California at Los Angeles, *VAS* visual analog scale

### Results of reliability analysis

Measurement of inter-observer reliability of radiographic images resulted in an ICC value of 0.945 for CSA, 0.938 for AI, and 0.908 for GI (Table 3).

**Table 3:** ICCs for inter-observer and intra-observer reliability

	CSA	AI	GI
ICC	.945	.938	.908

*CSA* critical shoulder angle, *AI* acromial index, *GI* glenoid inclination, *ICC* Intraclass correlation coefficient

## Discussion

Prior to this study, many reports have been published discussing the effects of differences in geometry of the scapula such as the CSA, AI, or GI on RCTs and shoulder osteoarthritis [20–23]. In these studies, the magnitude of the measurements was shown to greatly affect the incidence of shoulder joint disease. However, the long-term effects of the value of the CSA on the healing of RCT repairs have not been studied.

The mean CSA of all patients was 35.79° ± 3.59° in our study, similar to that measured in previously published literature [24, 25]. Garcia et al. [26] used ultrasound to establish the effect of high CSA values on postoperative repair of rotator cuffs. The results demonstrated that large angles significantly increased the risk of tendon tear recurrence after repair of the rotator cuff. In our study, patients with CSA > 38° had a threefold higher risk of rotator cuff re-tear than patients with a CSA < 38°. This indicates that a high CSA increases the risk of re-tear after a single supraspinatus tear. A biomechanical study by Gerber et al. [27] demonstrated that high CSA can cause an overload of the supraspinatus tendon during active abduction of the shoulder joint, which is consistent with our study. Because of the effect of a high CSA on rotator cuff injury, researchers have conducted cadaveric studies that reduced the lateral extension of the CSA by performing a lateral acromioplasty to reduce lateral extension of the acromion [28, 29]. In our study, the acromion did not undergo lateral resection, so patients with a high CSA were still subjected to a high supraspinatus load after surgery. This maybe a good explanation for why the re-tear rate of the rotator cuff in the high CSA patients in our study was lower than that in the low CSA patients.

Many studies have reported the relationship between AI and degenerative cuff tears, with a large AI increasing the risk of RCT [16, 30, 31]. In our study, mean AI was 0.72 ± 0.05. There was a significant difference between the AI and re-tear groups, indicating that the value of AI affected the recovery from RCT repairs, similar to the results of Zumstein et al. [32]. Ames et al. [14] followed up for 2 years after RCT surgery, finding that high AI reduced patient satisfaction. These results were similar to Lee et al. [33]. In our study, there was no significant correlation between AI and postoperative function.

GI has been shown to be a risk factor in supraspinatus tears [34–36]. The present study showed that mean GI was significantly different in the intact and re-tear groups (*p* < 0.05). For a GI > 14°, the risk of patient rotator cuff re-tear was five times higher. Gerber et al. [13] reported similar findings, in that high GI significantly increased the risk of re-tear after rotator cuff repair.

In our 2-year follow-up, patients completed a questionnaire and underwent shoulder function tests (CCA, ASES, UCLA, VAS). Our results indicated that there was no significant correlation in patient’s postoperative function and CSA, AI, or GI. Lee et al. [33] also compared the relationship between CSA, AI, and postoperative shoulder function, with similar results to the present study, with no significant relationship between any parameter. In addition, our results showed that there was no significant relationship between the integrity of the rotator cuff and shoulder joint function following surgery. Patient rotator cuff re-tear does not necessarily lead to functional deterioration. Millett et al. [37] demonstrated that patients with RCTs are not necessarily symptomatic, sometimes taking more than 2 years for symptoms to develop. Further follow-up studies are required to ascertain whether rotator cuff re-tears further affect shoulder function.

There are some limitations in our research. Firstly, the sample size was small, so the results may be biased. Secondly, even though the imaging measurements were reliable, as this was a retrospective study, the angle of the shoulder joint in the X-rays may not have been correctly positioned. Finally, MRI and shoulder function were only tested 2 years after surgery, and no short-term tests were performed, so the dynamic relationship between shoulder function and rotator cuff repair could not be fully evaluated.

## Conclusions

This study demonstrated that although higher CSA increased the risk of rotator cuff re-tear, there was no significant effect on shoulder function after rotator cuff repair. Similarly, the magnitude of AI and GI did not affect shoulder joint function after rotator cuff repairs. It can be seen that the specific geometry of the scapula affects the repair of the rotator cuff after shoulder surgery, but not the recovery of shoulder function.

## Data Availability

Not applicable

## Abbreviations

AI: Acromial index
ASES: American Shoulder and Elbow Surgeon
CSA: Critical shoulder angle
CSS: Constant Shoulder Score
GI: Glenoid inclination
ICC: Intraclass correlation coefficient
MRI: Magnetic resonance imaging
RCT: Rotator cuff tears
UCLA: University of California at Los Angeles
VAS: Visual analog scale
